# The Ten Laws of Health

**Published:** 1872-10

**Authors:** 


*The Ten Laws of Health; or how Disease is produced and can be prevented.* By J. R. BLACK, M. D. Philadelphia: J. B. Lippincott & Co., 1872. Buffalo: H. H. Otis.

This is a work which is designed more for the general reader than for the professional man. Yet from the hasty glance we have been able to bestow upon it we see many things which would be of interest and value to the medical practitioner, which doubtless would be increased on a more thorough examination. The introductory chapter treats of Disease, why it arises; with some considerations in reference to its preventability. The ten laws are: Breathing a pure air, Adequate and wholesome food and drink, Adequate outdoor exercise, Adequate and unconstraining covering for the body, The exercise of the sexual function, Habitation in the climate for which the constitution is adapted, Pursuits which do not cramp or overtax any part of the body, or subject it to irritating and poisonous substances, Personal cleanliness, Tranquil state of the mind and adequate rest and sleep. No intermarriage of near blood relations. Under each law are some remarks upon the results of violation, and also upon the mode of observing the law.

The laws laid down by the author would without doubt if followed be the cause of preventing much disease among the human family. The author is, we think, somewhat too sanguine in many of his statements, yet with proper allowance for some of his expected results, the book will be found correct and will impart much valuable information both to the professional and unprofessional reader.

